# Supplementary material for: Molecular Epidemic Characteristics and Genetic Evolution of Porcine Circovirus Type 2 in Henan, China
Source: Vet Sci. 2025 Apr 7;12(4):343. doi: 10.3390/vetsci12040343 (PMC12031534; doi:10.3390/vetsci12040343)
Supplement: Supplementary file 1 [file vetsci-12-00343-s001.zip › vetsci-3552675-supplementary.pdf]

Supplementary Tables

Table S1. Background of clinical cases of porcine circovirus-associated diseases from pig farms distributed in Henan Province, China.

No.	Pig Farms	Geographical Location	Age Group for Sample Collection	Pig No.	Organ Sampled	Main Clinical Sign	Morbidity	Year of collection
1	HNZK	Zhoukou	Weaned	3 000	Inguinal lymph node, spleen	Wasting, weight loss	5.00%	2020
2	HNXX1	Xinxiang	Weaned	5 500	Inguinal lymph node, spleen	Wasting, weight loss	10.00%	2020
3	HNNY1	Nanyang	Weaned	4 500	Inguinal lymph node, spleen	Respiratory distress, dyspnea	12.00%	2020
4	HNJZ1	Jiaozuo	Weaned	2 000	Inguinal lymph node, spleen	Wasting, weight loss	8.00%	2020
5	HNXC1	Xuchang	Weaned	6 000	Inguinal lymph node, spleen	Respiratory distress, dyspnea	10.00%	2020
6	HNAY1	Anyang	Finishing	4 000	Inguinal lymph node, spleen	Wasting, weight loss	15.00%	2020
7	HNXX2	Xinxiang	Weaned	3 600	Inguinal lymph node, spleen	No obvious clinical signs	5.5%	2020
8	HNHB	Hebi	Weaned	4 700	Inguinal lymph node, spleen	Wasting, weight loss	10.00%	2020
9	HNAY2	Anyang	Finishing	5 000	Inguinal lymph node, spleen	Wasting, weight loss	8.00%	2020
10	HNJY	Jiyuan	Finishing	3 500	Inguinal lymph node, spleen	Dark red papules and macules on skin	8.00%	2020
11	HNAY3	Anyang	Finishing	4 000	Inguinal lymph node, spleen	Dark red papules and macules on skin	6.00%	2021
12	HNZZ	Zhengzhou	Suckling	6 500	Inguinal lymph node, spleen	Respiratory distress, dyspnea	4.00%	2021
13	HNPDS	Pingdingshan	Weaned	4 800	Inguinal lymph node, spleen	No obvious clinical signs	5.00%	2021
14	HNSQ1	Shangqiu	Weaned	3 000	Inguinal lymph node, spleen	Dark red papules and macules on skin	0.80%	2021
15	HNLH	Luohe	Weaned	1 800	Inguinal lymph node, spleen	Dark red papules and macules on skin	0.50%	2021
16	HNNY2	Nanyang	Suckling	3 200	Inguinal lymph node, spleen	Respiratory distress, dyspnea	5.00%	2021

17	HNZMD1	Zhumadian	Suckling	6 500	Inguinal lymph node, spleen	Respiratory distress, dyspnea	5.40%	2021
18	HNSMX1	Sanmenxia	Finishing	3 600	Inguinal lymph node, spleen	Dark red papules and macules on skin	0.50%	2021
19	HNXX3	Xinxiang	Finishing	4 000	Inguinal lymph node, spleen	Decreased average daily gain	2.00%	2022
20	HNAY4	Anyang	Weaned	3 800	Inguinal lymph node, spleen	Wasting, weight loss	5.50%	2022
21	HNXY	Xinyang	Weaned	4 600	Inguinal lymph node, spleen	No obvious clinical signs	5.00%	2022
22	HNXC2	Xuchang	Weaned	1 800	Inguinal lymph node, spleen	Wasting, weight loss	10.00%	2022
23	HNLY1	Luoyang	Finishing	2 600	Inguinal lymph node, spleen	Respiratory distress, dyspnea	5.00%	2022
24	HNNY3	Nanyang	Weaned	4 600	Inguinal lymph node, spleen	Dark red papules and macules on skin	0.50%	2022
25	HNFQ	Xinxiang	Weaned	3 000	Inguinal lymph node, spleen	No obvious clinical signs	4.00%	2022
26	HNLY2	Luoyang	Weaned	3 600	Inguinal lymph node, spleen	Respiratory distress, dyspnea	5.00%	2022
27	HNJZ2	Jiaozuo	Weaned	4 000	Inguinal lymph node, spleen	Dark red papules and macules on skin	0.70%	2023
28	HNZMD2	Zhumadian	Suckling	1 800	Inguinal lymph node, spleen	Respiratory distress, dyspnea	20.00%	2023
29	HNXC3	Xuchang	Suckling	2 000	Inguinal lymph node, spleen	Respiratory distress, dyspnea	15.00%	2023
30	HNSQ2	Shangqiu	Weaned	1 600	Inguinal lymph node, spleen	Respiratory distress, dyspnea	5.00%	2023
31	HNNY4	Nanyang	Weaned	2 400	Inguinal lymph node, spleen	Dark red papules and macules on skin	0.50%	2023
32	HNAY5	Anyang	Weaned	1 000	Inguinal lymph node, spleen	Dark red papules and macules on skin	0.80%	2023
33	HNSMX2	Sanmenxia	Suckling	850	Inguinal lymph node, spleen	Respiratory distress, dyspnea	4.00%	2023
34	HNAY6	Anyang	Weaned	1 200	Inguinal lymph node, spleen	Wasting, weight loss	5.00%	2023

Table S2. Accession numbers of PCV2 strains isolated in Henan Province and other reference PCV2 strains used in this study.

Year	Accession No.						Seq. No.
2004	EU346945.1						1
2005	AY969004.1						1
2007	EU418627.1	EU418626.1	EU647557.1	EU521707.1	EU521708.1	EU521709.1	7
	EU555439.1						
2008	FJ870974.1	FJ870976.1	FJ440338.1	EU780073.1	EU780074.1	EU656143.1	6
2009	HQ693092.1	GU938302.1	GU450328.1				3
2010	HQ693093.1	HQ650833.1					2
2011	JN615187.1	JN119255.1	JN119256.1	JN119257.1	JF928002.1	JF928003.1	10
	JF928004.1	JF928005.1	JF928006.1	JF899334.1			
2012	KC753768.1	KC753769.1	KC753770.1	KC753771.1	KC753772.1	KC733435.1	9
	KC684978.1	JX534236.1	JX534237.1				
2013	KU960935.1	KU960938.1	KF926650.1	KC821781.1	KC821782.1	KC821783.1	8
	KC821784.1	KC821785.1					
2014	KU960929.1	KU960930.1	KU960936.1	KU960932.1	KM067384.1	KM067385.1	8
	KM035761.1	KM035762.1					
2015	MK604512.1	MK604497.1	KU960931.1	KU960933.1	KU960934.1	KU960937.1	11
	MK604500.1	MK604484.1	KU960939.1	KU960940.1	KU960941.1		
2016	MH055401.1	MK604492.1	MK604488.1	MK604490.1	MH055402.1	MK604479.1	33

	MH055403.1	MH055404.1	MH055405.1	MH055406.1	MK604495.1	MH055407.1	
	MH055408.1	MH055409.1	MK604501.1	MK604503.1	MK604505.1	MK604506.1	
	MK604508.1	MK604509.1	MK604510.1	MK604511.1	MK604498.1	MK604513.1	
	MK604514.1	MH055410.1	MH055411.1	MH055412.1	MG491307.1	MG491308.1	
	MG517443.1	MH046794.1	MH046795.1				
2017	MK604499.1	MK604507.1	MK604486.1	MT423827.1	MK604502.1	MK604483.1	25
	MK604491.1	MK604493.1	MK604504.1	MK604515.1	MH046838.1	MH046839.1	
	MH046840.1	MH059556.1	MH059557.1	MH059558.1	MH059559.1	MH059560.1	
	MH059561.1	MH059562.1	MH059563.1	MH059564.1	MH059565.1	MH059566.1	
	MH059567.1						
2018	MW117136.1	MK604480.1	MK604481.1	MK604482.1	MK604487.1	MK604489.1	8
	MK604494.1	MH323413.1					
2019	MT920415.1	MT876209.1	MT876210.1	MT876211.1	MT876212.1	MT876213.1	8
	MT876214.1	MW538944.1					
	AY686763.1	JQ806749.1	HQ402903.1	AY579893.1	HM038031.1	HM038030.1	18
	HM038032.1	EU257511.1	FJ598044.1	HM641752.1	HM038034.1	EU148504.1	
	EU148505.1	KT795287.1	KT795290.1	JX099786.1	FJ998185.1	KC800644.1	

Supplementary Figures

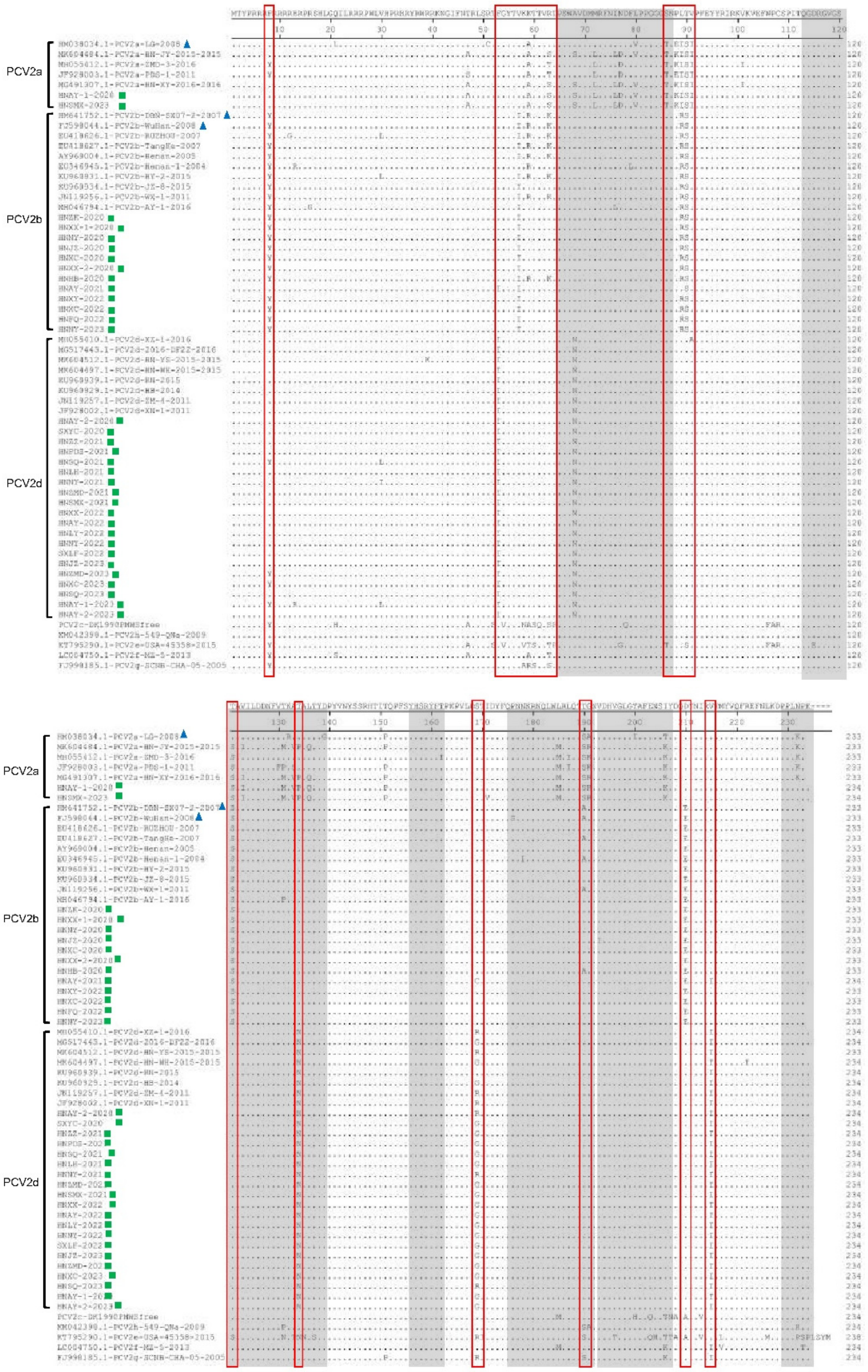

Figure S1. Amino acid sequence alignment of the PCV2 Cap protein.
